# Excess hospitalizations and mortality associated with seasonal influenza in Portugal, 2008–2018

**DOI:** 10.1186/s12879-022-07713-8

**Published:** 2022-09-07

**Authors:** Filipe Froes, Mafalda Carmo, Hugo Lopes, Geoffray Bizouard, Catarina Gomes, Margarida Martins, Hélène Bricout, Caroline de Courville, Jaime Correia de Sousa, Carlos Rabaçal, João F. Raposo, Carlos Robalo Cordeiro

**Affiliations:** 1 ICU, Thorax Department, Centro Hospitalar Universitário Lisboa Norte, Av. Prof. Egas Moniz MB, 1649-028 Lisbon, Portugal; 2 IQVIA, Barcelona, Spain; 3 IQVIA, Lisbon, Portugal; 4 NOVA National School of Public Health, Public Health Research Centre, Universidade NOVA de Lisboa, Lisbon, Portugal; 5 Comprehensive Health Research Center (CHRC)—Universidade NOVA de Lisboa, Lisbon, Portugal; 6 IQVIA, Paris, France; 7 Sanofi, Lisbon, Portugal; 8 Sanofi, Lyon, France; 9 Life and Health Sciences Research Institute (ICVS), School of Medicine, University of Minho, Braga, Portugal; 10 ICVS/3B’s, PT Government Associate Laboratory, Braga/Guimarães, Portugal; 11 Vila Franca de Xira Hospital, Lisbon, Portugal; 12 APDP and NOVA Medical School, Universidade NOVA de Lisboa, Lisbon, Portugal; 13 Pulmonology Department, Coimbra University Hospital, University of Coimbra, Coimbra, Portugal

**Keywords:** Influenza, Burden, Excess, Mortality, Hospitalization, Cardiovascular, Respiratory, Portugal

## Abstract

**Background:**

Influenza can have a domino effect, triggering severe conditions and leading to hospitalization or even death. Since influenza testing is not routinely performed, statistical modeling techniques are increasingly being used to estimate annual hospitalizations and deaths associated with influenza, to overcome the known underestimation from registers coded with influenza-specific diagnosis. The aim of this study was to estimate the clinical and economic burden of severe influenza in Portugal.

**Methods:**

The study comprised ten epidemic seasons (2008/09–2017/18) and used two approaches: (i) a direct method of estimating the seasonal influenza hospitalization incidence, based on the number of National Health Service hospitalizations with influenza-specific International Classification of Diseases (ICD) codes (ICD-9: 487–488; ICD-10: J09-J11), as primary or secondary diagnosis; (ii) an indirect method of estimating excess hospitalizations and deaths using broader groups of ICD codes in time-series models, computed for six age groups and four groups of diagnoses: pneumonia or influenza (ICD-9: 480–488, 517.1; ICD-10: J09–J18), respiratory (ICD-9: 460–519; ICD-10: J00–J99), respiratory or cardiovascular (R&C, ICD-9: 390–459, 460–519; ICD-10: I00–I99, J00–J99), and all-cause. Means are reported excluding the H1N1pdm09 pandemic (2009/10).

**Results:**

The mean number of hospitalizations coded as due to influenza per season was 1,207, resulting in 11.6 cases per 100,000 people. The mean direct annual cost of these hospitalizations was €3.9 million, of which 78.6% was generated by patients with comorbidities. Mean annual influenza-associated R&C hospitalizations were estimated at 5356 (min: 456; max: 8776), corresponding to 51.5 cases per 100,000 (95% CI: 40.9–62.0) for all age groups and 199.6 (95% CI: 163.9–235.8) for the population aged ≥ 65 years. The mean direct annual cost of the estimated excess R&C hospitalizations was €15.2 million for all age groups and €12.8 million for the population aged ≥ 65 years. Mean annual influenza-associated all-cause deaths per 100,000 people were estimated at 22.7 for all age groups.

**Conclusions:**

The study findings suggest that there is an under-detection of influenza in the Portuguese population. A high burden of severe influenza remains to be addressed, not only in the elderly population but also in younger people.

**Supplementary Information:**

The online version contains supplementary material available at 10.1186/s12879-022-07713-8.

## Background

Influenza is a well-known acute infectious viral respiratory disease, responsible for seasonal epidemic outbreaks. While most people experience only mild symptoms, influenza can have a domino effect, triggering severe conditions and leading to hospitalization or even death—either directly associated with influenza or related to decompensation from other existing illnesses or frailty [1–3]. Annual vaccination is recommended by the World Health Organization (WHO) for health care workers and at-risk individuals, namely children aged 6–59 months,^1^ pregnant women, elderly individuals, individuals aged > 6 months with certain chronic medical conditions, residents of nursing homes, and the disabled [4].

Excess morbidity and mortality studies using regression techniques to estimate the influenza-associated disease burden have been successfully used worldwide in an effort to overcome the challenges of inadequate influenza laboratory testing and coding practices [5]. In Portugal, fewer than 200 deaths are registered as being due to influenza per year, whereas all-cause excess mortality estimates published by the National Sentinel Surveillance System have surpassed the 3000 mark [6]. Data on influenza-associated excess hospitalization in Portugal from seasons 1998/99 to 2014/15 are also available and suggest that 80% of analyzed influenza epidemics were associated with excess hospitalization [7]. However, the study covers only pneumonia or influenza (P&I) hospitalizations, whereas evidence increasingly supports an analysis of the broader impact of influenza, as it leads not only to respiratory complications but also to exacerbation of underlying chronic conditions, functional decline, vulnerability to secondary bacterial infections, as well as rare complications affecting other organ systems (e.g., nervous, cardiovascular) [2]. The study also reports variable excess P&I hospitalization patterns according to age group and the circulating virus [7], thus highlighting the need to continuously monitor the burden of influenza, as the intensity and severity of influenza epidemics may vary according to specific strains of influenza viruses and vaccination coverage rates—among other variables [2].

There is a need for updated estimates on the influenza-associated excess hospitalization and mortality in Portugal, taking into account the impact of influenza on other respiratory and non-respiratory complications, and comparing these estimates to those obtained through direct analysis of influenza-coded cases.

This study aims to estimate the clinical and economic burden of severe influenza in Portugal from season 2008/09 to season 2017/18 across distinct age groups and, particularly, to: (i) describe and estimate the cumulative seasonal incidence of influenza-coded National Health Service (NHS) hospitalizations by age group and comorbidities; (ii) estimate the number of excess hospitalizations and deaths during influenza epidemics that are influenza-associated, considering broader groups of diagnoses.

## Methods

The Burden of Acute Respiratory Infections (BARI) study is a multidimensional real-world evidence study assessing the clinical and economic burden of acute respiratory infections (influenza and respiratory syncytial virus) in Spain and Portugal. Here we report results for the burden of severe influenza in Portugal, measured through hospitalizations and deaths.

This publication employs the following two approaches: (i) a direct method of estimating seasonal influenza incidence, based on the number of NHS hospitalizations with influenza-specific International Classification of Diseases (ICD) codes; (ii) an indirect method of estimating excess hospitalizations and deaths using broader groups of ICD codes in time-series ecological models.

### Data sources

#### Hospital discharge data

Anonymized administrative data on hospitalizations (January 2008–December 2018) were provided by ACSS—*Administração Central do Sistema de Saúde* (Health System Central Administration), which collects administrative and clinical data for all hospitalization episodes in Portuguese public hospitals, including information on diagnoses and procedures performed during hospital stay, which are coded using the ICD-9-CM and ICD-10-CM/PCS. These data were also used to describe the in-hospital case fatality risk based on discharge status, corresponding to the hospitalization episodes coded as due to influenza that resulted in in-hospital death during the respective episode.

#### Death certificate data

For analysis of influenza-associated excess mortality, data on daily deaths listed in the death certificate per age group and cause were obtained from the *Instituto Nacional de Estatística* (INE, National Statistics Institute) in July 2020. The data were aggregated into weekly counts [8]. The coding for primary death from the death certificate was used.

#### Influenza activity data

The primary predictor of influenza excess hospitalizations and deaths was the overall weekly incidence rate of influenza-like-illness (ILI), which was obtained from the *Instituto Nacional de Saúde Doutor Ricardo Jorge* (INSA, National Institute of Health Doutor Ricardo Jorge) in July 2020 [9]. This rate is estimated by the INSA based on data collected by the Portuguese General Practitioners Sentinel Network (*Rede Médicos Sentinela*) [10].

#### Demographic data

Finally, in order to compute rates of cases per 100,000 people, data on age-specific annual resident population estimates were downloaded from INE’s website [8].

### Statistical analysis

An ecological approach was used to estimate the number of influenza-associated excess hospitalizations, deaths, and hospitalization costs by using cyclic regression models explicitly modeling weekly morbidity and mortality data against weekly indicators of influenza activity (moving average, broken down by season, with a one-week lag for hospitalizations and 3-week lag for deaths).

Different Poisson cyclic models (time series) were used, where age- and cause-specific hospitalization and mortality data were explained by the ILI incidence [5, 11, 12], as well as time trends and seasonal terms, using a log link [13, 14]. A stepwise selection method was used to identify significant time trends and seasonal terms in the age- and cause-specific models.

Baseline hospitalization and mortality data were calculated from Poisson models as model-expected values when the ILI variable was set to zero. The weekly number of influenza-associated excess hospitalizations and mortality was estimated as the difference between expected hospitalizations and deaths estimated by the Poisson model, incorporating the ILI incidence rate as an indicator of influenza activity and the number of hospitalizations and deaths estimated by the baseline model not taking into account this indicator. The number of influenza-associated hospitalizations or deaths was defined for each epidemic season by the sum of the weekly excesses. The seasons were defined from September to June of each year, as defined for the Northern Hemisphere.

The performance of the model was measured by the correlation between the values predicted by the model and those observed using Pearson’s correlation. The mean absolute percentage error (MAPE) was also computed as the average, for all weeks, of the percentage difference between predicted and observed values.

For the excess hospitalization and mortality analysis, the hospitalizations and deaths were organized into four categories according to cause (P&I, respiratory, respiratory, or cardiovascular, and all-cause). Regarding age groups, the population was stratified thus: 0–4 years old, 5–18, 19–49, 50–64, 65–74, ≥ 75; and ≥ 65 years old.

Confidence intervals of 95% were calculated for the estimated influenza-associated excess hospitalizations and deaths by cause and age group.

The estimates of influenza-associated excess hospitalization and mortality were compared to the numbers of primary or secondary influenza‐specific diagnoses captured from NHS hospital discharge records, as well as fatalities observed during these episodes.

Cases per 100,000 people were computed by dividing the estimated influenza cases by the Portuguese population in the respective age group.

Means are reported only for nine seasons, excluding the H1N1pdm09 pandemic (2009/10).

### Case definition

#### Hospitalizations coded as due to influenza

Data were extracted from administrative databases from 2008 to 2018 that contained all NHS hospitalization records in mainland Portugal. Influenza episodes were defined as those coded with ICD-9 487 or 488; or ICD-10 J09, J10, or J11 in any primary or secondary diagnosis field. Total hospitalizations excluded admissions related to routine birth, planned activity, and those in which the primary diagnosis was related to musculoskeletal, alcoholic, or mental disease. Additional diagnostic information during the influenza episode was also used to identify individuals who had at least one medical condition regarded as a risk factor for severe influenza (Additional file 1: Table S1) in any primary or secondary diagnosis field, considering the following conditions: pregnancy, diabetes mellitus, respiratory or lung disease, cardiovascular disease, immunocompromised, chronic liver disease, and chronic kidney disease.

### Excess hospitalization and mortality

Influenza-associated excess hospitalization was computed for four groups of diagnoses [15, 16], according to the primary diagnosis, namely pneumonia or influenza (P&I, ICD-9: 480–488, 517.1; or ICD-10: J09–J18), respiratory (R, ICD-9: 460–519; or ICD-10: J00–J99), respiratory or cardiovascular (R&C, ICD-9: 390–459, 460–519; or ICD-10: I00–I99, J00–J99), and all-cause (any ICD-9/10 diagnosis). Influenza-associated excess mortality was computed for the same groups of diagnoses according to the cause of death listed on the death certificate.

### Cost estimation

#### Hospitalizations coded as due to influenza

Only direct costs were estimated using a diagnosis-related group (DRG)-based budget allocation model. Hospitalization costs were computed by multiplying each cost weight, considering the DRG of each episode, with the Portuguese fixed cost multiplier and funding price applicable for the 2018 year, as defined by the ACSS.

In Portugal, although there is no detailed cost information at the patient level [17, 18] and the provision of health care is mostly public, with no need to create routines or billing procedures in the definition of costs per DRG, the Maryland matrix was applied [17]. This matrix makes the relative correspondence according to North American standards of care provision between the care provided during the hospitalization episode and assigns relative weights that reflect the costs per service for each DRG [18]. Although this methodology has a few limitations, such as the assumption that the pattern of resource use is similar to the American one, according to Bentes et al., it seems to be the most cost-effective method to identify costs per DRG, having been in use since the implementation of DRG in Portugal in the 1980s [19, 20].

### Excess hospitalization

The cost of excess hospitalization was computed by multiplying the number of estimated excess hospitalizations by the mean cost per hospitalization, by cause and age group.

### Ethical considerations

The study was conducted following the ethical principles of the Declaration of Helsinki and as per local regulations, including privacy laws. Data were provided anonymized and may be used for research purposes without the approval of an ethics committee or informed consent. In addition, the protocol of the BARI study was validated by a panel of clinical experts, classified by the Agency of Medicines and Medical Devices (AEMPS) as an observational study and approved by the Ethics Committee of Hospital Clinic de Barcelona (HCB/2020/1132), who waived the need for participant consent.

## Results

### Descriptive statistics for unmodeled hospitalizations and deaths

In 2018, Portugal had a resident population of 10.3 million, of which 21.8% were aged ≥ 65 years [21]. During the study period (10 seasons), a total of 2.2 million R&C hospitalizations were registered in Portuguese public hospitals, with 1.4 million (65.2%) in patients aged ≥ 65 years. Respiratory causes accounted for 45.0% of total R&C hospitalizations. During the same period, a total of 1.1 million people died in Portugal, of whom 0.9 million (83.7%) were aged ≥ 65 years.

### Influenza epidemiologic burden

#### Hospitalizations coded as due to influenza

A total of 13,629 influenza hospitalizations were registered between seasons 2008/2009 and 2017/2018, of which 6567 (48.2%) were registered during the last three analyzed seasons. Excluding season 2009/10, which was affected by the H1N1 pandemic, the mean annual number of hospitalizations per season was 1207 (Table 1), corresponding to 11.6 cases per 100,000 people^2^ (Table 2). The peak of annual hospitalizations was observed in season 2017/18 (3007; 29.3 per 100,000).

**Table 1:** Number of hospitalizations coded as due to influenza by age groups and epidemic season in Portuguese public hospitals between 2008/2009 and 2017/2018

Season	Age groups (in years)							
	0–4	5–18	19–49	50–64	65–74	≥ 75	≥ 65	All ages
2008/2009	117	36	59	44	49	95	144	400
2009/2010	617	429	1070	393	146	115	261	2770
2010/2011	158	57	232	165	68	81	149	761
2011/2012	128	26	77	49	67	134	201	481
2012/2013	122	56	194	187	86	112	198	757
2013/2014	180	43	233	209	108	169	277	942
2014/2015	154	69	159	152	155	262	417	951
2015/2016	340	89	299	381	240	273	513	1622
2016/2017	170	55	148	261	322	982	1304	1938
2017/2018	333	130	256	477	491	1320	1811	3007
9-year mean^a^	189	62	184	214	176	381	557	1207
3-year mean^b^	281	91	234	373	351	858	1209	2189

^a^Includes the following seasons: 2008/2009, 2010/2011, 2011/2012, 2012/2013, 2013/2014, 2014/2015, 2015/2016, 2016/2017, 2017/2018. Excludes season 2009/10, since it was affected by the 2009 influenza A (H1N1) pandemic

^b^Includes the most recent seasons, namely 2015/2016, 2016/2017, 2017/2018

**Table 2:** Number of hospitalizations coded as due to influenza by age group and epidemic season per 100,000 people in Portuguese public hospitals between 2008/2009 and 2017/2018

Season	Age groups (in years)							
	0–4	5–18	19–49	50–64	65–74	≥ 75	≥ 65	All ages
2008/2009	22.8	2.3	1.3	2.2	4.8	10.4	7.5	3.8
2009/2010	123.5	27.8	23.6	19.4	14.2	12.1	13.2	26.2
2010/2011	32.2	3.7	5.2	8.1	6.6	8.3	7.4	7.2
2011/2012	26.6	1.7	1.8	2.4	6.5	13.5	9.9	4.6
2012/2013	26.3	3.7	4.5	9.0	8.1	11.0	9.6	7.3
2013/2014	40.0	2.9	5.5	10.0	10.1	16.4	13.2	9.1
2014/2015	35.3	4.7	3.8	7.2	14.2	25.0	19.5	9.2
2015/2016	79.4	6.1	7.3	17.9	21.5	25.8	23.6	15.7
2016/2017	39.9	3.8	3.6	12.2	28.2	91.6	58.9	18.8
2017/2018	77.4	9.2	6.3	22.2	42.5	121.4	80.7	29.3
9-year mean^a^	41.3	4.2	4.3	10.2	16.3	37.3	26.5	11.6
3-year mean^b^	65.6	6.3	5.7	17.5	30.8	80.0	54.7	21.3

^a^Includes the following seasons: 2008/2009, 2010/2011, 2011/2012, 2012/2013, 2013/2014, 2014/2015, 2015/2016, 2016/2017, 2017/2018. Excludes season 2009/10, since it was affected by the 2009 influenza A (H1N1) pandemic

^b^Includes the most recent seasons, namely 2015/2016, 2016/2017, 2017/2018

Patients aged ≥ 65 years contributed to 46.2% of influenza hospitalizations^2^ (Table 3), with a mean of 26.5 cases per 100,000 over nine seasons, reaching 80.7 cases per 100,000 (60.2% of hospitalizations) in the 2017/18 season. Patients with comorbidities accounted for 65.6% of hospitalizations, with 62.0% aged ≥ 65 years^2^.

**Table 3:** Summary of the characteristics of the hospitalizations for influenza by age group and existence of comorbidities in Portuguese public hospitals between 2008/2009 and 2017/2018

Variable	Age groups (in years)									With comorbidities	Without comorbidities	
	0–4	5–18	19–49	50–64	65–74	≥ 75	≥ 65	All ages	< 65	≥ 65	< 65	≥ 65
Share of hospitalizations (%)												
9-year mean^a^	15.7	5.2	15.3	17.7	14.6	31.6	46.2	100.0	24.9	40.6	28.9	5.5
3-year mean^b^	12.8	4.2	10.7	17.0	16.0	39.2	55.2	100.0	21.5	48.2	23.2	7.0
[Min; Max]	[8.8; 29.3]	[2.8; 15.5]	[7.6; 38.6]	[10.2; 24.7]	[5.3; 16.6]	[4.2; 50.7]	[9.4; 67.3]	[100; 100]	[15.8; 36.1]	[7.7; 59.6]	[15.2; 57.3]	[1.7; 8.6]
Influenza as primary diagnosis (%)												
9-years mean^a^	73.6	72.7	63.5	62.5	60.2	64.6	63.2	65.2	58.6	61.2	74.2	77.9
3-year mean^b^	74.5	76.3	65.6	65.1	64.3	68.2	67.1	67.9	61.9	65.2	75.5	79.6
[Min; Max]	[66.2; 86.3]	[62.8; 84.6]	[42.9; 84.3]	[36.7; 80.2]	[37.3; 74.7]	[42.1; 73]	[42.4; 73.9]	[52.6; 82.4]	[35.6; 79.1]	[33.9; 71.5]	[65.7; 85.8]	[62.1; 85.1]
LoS (in days)												
9-year mean^a^	6.1	6.0	10.2	13.2	12.6	11.8	12.0	10.7	12.4	12.4	7.1	9.5
3-year mean^b^	6.0	5.7	10.8	12.1	12.5	12.2	12.3	11.0	11.8	12.7	7.2	9.4
[Min; Max]^c^	[4.4; 7]	[4.2; 7.3]	[4.9; 11.5]	[9.3; 18]	[9.6; 14.2]	[9.6; 12.5]	[10; 12.7]	[7.1; 11.4]	[8.7; 15.5]	[10.1; 13.1]	[4.8; 8]	[7.5; 13.4]
In-hospital case fatality risk (%)												
9-year mean^a^	0.2	0.9	3.4	6.2	7.4	10.5	9.5	6.1	5.3	9.9	1.3	6.8
3-year mean^b^	0.1	1.5	4.1	5.6	7.5	10.4	9.5	6.7	5.7	9.9	1.1	7.0
[Min; Max]^c^	[0; 0.8]	[0; 2.2]	[0; 4.7]	[2; 9.1]	[0; 11.8]	[5.2; 12.5]	[3.4; 11.4]	[2.4; 7.8]	[1; 6.5]	[1.9; 11.2]	[0.4; 2.7]	[0; 15.8]
Use of supplemental oxygen (%)												
9-year mean^a^	34.3	18.4	38.4	45.4	44.2	49.1	47.5	42.2	44.1	47.9	31.9	45.2
3-year mean^b^	36.7	16.1	36.8	45.8	43.9	47.8	46.7	42.9	43.6	46.5	33.3	47.6
[Min; Max]^c^	[17.8; 41.8]	[2.8; 30.8]	[6.8; 48.8]	[20.5; 54.6]	[36.7; 52.9]	[42.9; 60.8]	[44.4; 57.1]	[24.8; 49]	[22.2; 52.9]	[44.2; 57.5]	[10.9; 38.2]	[26.3; 63]
Use of non-invasive mechanical ventilation (%)												
9-year mean^a^	3.1	4.1	5.7	11.6	13.3	11.9	12.3	9.3	10.6	13.4	3.4	4.3
3-year mean^b^	3.6	5.5	7.5	12.6	14.1	12.6	13.0	10.8	12.3	14.1	4.3	5.2
[Min; Max]^c^	[0.6; 5.1]	[0; 9]	[0; 9.8]	[4.1; 18.4]	[0; 16.1]	[1.1; 14.3]	[0.7; 14.3]	[1; 12.1]	[2.7; 13.7]	[0.8; 15.7]	[0.5; 5.2]	[0; 6.5]
Use of invasive mechanical ventilation (%)												
9-year mean^a^	2.4	4.3	12.3	17.4	13.9	6.6	8.9	9.7	15.8	9.5	5.6	4.5
3-year mean^b^	2.3	6.2	12.9	15.5	13.2	6.5	8.5	9.3	15.8	9.2	5.0	3.5
[Min; Max]	[0.9; 4.9]	[0; 9.1]	[0; 16.4]	[2.3; 24.9]	[5.5; 19.8]	[1.1; 9.8]	[3.5; 14.1]	[1.8; 14.3]	[3.2; 20]	[4.2; 16]	[0; 10.1]	[0; 27.3]
Discharged to other healthcare service^d^ (%)												
9-year mean^a^	2.2	3.4	6.3	7.7	5.6	4.5	4.8	5.1	6.6	5.0	4.2	3.8
3-year mean^b^	2.1	3.6	7.0	7.5	4.8	4.3	4.4	4.9	6.6	4.7	4.5	2.6
[Min; Max]^c^	[0.8; 5.5]	[0; 5.8]	[0; 8.7]	[2; 12.3]	[2.5; 10.2]	[1.1; 7.1]	[3.5; 8.3]	[2.5; 6.8]	[1.6; 9]	[4; 7.1]	[1.6; 4.9]	[0; 22.7]
Mean hospitalization cost per patient^e^ (€)												
9-year mean^a^	1278	1855	3753	5074	4096	2974	3327	3302	4714	3485	2024	2164
3-year mean^b^	1174	2140	3805	4005	3885	3212	3174	3068	4411	3316	1614	2188
[Min; Max]^c^	[780; 2471]	[911; 2883]	[1441; 4647]	[3037;11030]	[2626; 6769]	[1621; 6154]	[2567; 5817]	[1791; 5994]	[2623; 8090]	[2718; 6514]	[917; 4228]	[1220; 4750]

^a^Includes the following seasons: 2008/2009, 2010/2011, 2011/2012, 2012/2013, 2013/2014, 2014/2015, 2015/2016, 2016/2017, 2017/2018. Excludes season 2009/10, since it was affected by the 2009 influenza A (H1N1) pandemic

^b^Includes the most recent seasons, namely 2015/2016, 2016/2017, 2017/2018

^c^Minimum and maximum values observed throughout the analyzed seasons for each age group

^d^Discharges to long-term hospital care, specialized care, post-hospital care, palliative care, transference to other institution with inpatient care or home care; ^e^ Includes > 1 influenza hospitalization/patient

The mean length of stay (LoS) stood at 10.7 days, increasing to 12.0 in patients aged ≥ 65 and to 12.4 in patients with comorbidities^2^. Considering only age, those between 50 and 65 years old presented the highest mean LoS (13.2 days) and use of invasive mechanical ventilation (in 17.4% of hospitalizations)^2^. The mean in-hospital case fatality risk was 6.1%^2^. In addition, patients were discharged to other health care services in 5.1% of hospitalizations^2^. These severity indicators are detailed by age group and presence of comorbidities in Table 3.

In two-thirds of hospitalizations (65.2%), influenza was the primary discharge diagnosis^2^. This percentage was higher in the pediatric population and in people without comorbidities, regardless of age (Table 3).

### Influenza-associated excess respiratory or cardiovascular hospitalization

A total of 49,929 (95% Confidence interval (CI): 39,734; 60,724) influenza-associated excess R&C hospitalizations were estimated across the study period (10 seasons), with the maximum value observed in the 2016/17 season (8776). Mean annual influenza-associated R&C hospitalizations over nine seasons were estimated at 5356^2^. Over nine seasons, the mean annual excess R&C hospitalization rate per 100,000 was estimated at 51.5 (95% CI: 40.9–62.0) for all age groups and 199.6 (95% CI: 163.9–235.8) for the population aged ≥ 65 years^2^. In the population aged < 65 years old, the highest mean annual excess R&C hospitalizations per 100,000 over nine seasons was estimated in those aged < 5 years old, at 43.2 (95% CI: − 124.8; 122.2), followed by those aged 50 to 64 years old, at 41.7 (95% CI: 31.5; 52.0). Absolute and relative annual influenza-associated excess R&C hospitalizations per age group are detailed in Tables 4 and 5. Results for excess R&C hospitalization are presented in this section, as they yielded the best model performance (Additional file 1: Table S2). Results for other groups of causes (P&I, respiratory, and all-cause) are presented in the Additional file 1: Table S4.

**Table 4:** Estimated number of influenza-associated excess respiratory or cardiovascular hospitalizations by age group and epidemic season in Portuguese public hospitals between 2008/2009 and 2017/2018

Season	Age groups (in years) 95% confidence interval							
	0–4	5–18	19–49	50–64	65–74	≥ 75	≥ 65	All ages
2008/2009	–	–	–	50 (− 141; 259)	847 (646; 1034)	3521 (2987; 3993)	4398 (3672; 5010)	4327 (3170; 5347)
2009/2010	93 (− 176; 371)	399 (323; 476)	1661 (1498; 1837)	657 (474; 837)	–	–	–	1730 (702; 2708)
2010/2011	–	11 (− 88; 112)	982 (764; 1205)	1201 (986; 1404)	653 (447; 861)	–	748 (45; 1486)	2824 (1706; 3959)
2011/2012	–	15 (− 70; 97)	405 (224; 602)	611 (412; 821)	1166 (972; 1349)	5567 (5041; 6148)	6806 (6105; 7511)	7212 (6142; 8277)
2012/2013	–	–	255 (50; 458)	546 (321; 778)	333 (96; 570)	548 (− 116; 1198)	1034 (215; 1891)	456 (− 804; 1676)
2013/2014	381 (165; 613)	–	762 (595; 930)	952 (736; 1162)	587 (392; 775)	1106 (533; 1643)	1756 (1049; 2429)	3376 (2361; 4389)
2014/2015	–	–	345 (199; 503)	878 (708; 1084)	1102 (928; 1276)	6204 (5700; 6726)	7446 (6852; 8088)	7988 (7093; 8941)
2015/2016	652 (377; 909)	277 (183; 366)	1098 (900; 1302)	1332 (1099; 1567)	697 (455; 921)	2279 (1593; 2978)	2974 (2129; 3886)	5845 (4639; 7057)
2016/2017	–	–	196 (25; 363)	905 (706; 1096)	919 (732; 1115)	6244 (5721; 6812)	7262 (6598; 7975)	8776 (7841; 9725)
2017/2018	662 (364; 942)	44 (− 49; 145)	437 (251; 636)	1391 (1139; 1631)	1173 (903; 1427)	3949 (3209; 4688)	5314 (4372; 6268)	7395 (6080; 8646)
9-year mean^a^	188 (− 604; 535)	39 (− 90; 112)	498 (288; 669)	874 (663; 1089)	831 (619; 1036)	3269 (2677; 3856)	4193 (3448; 4949)	5356 (4248; 6446)
3-year mean^b^	438 (118; 652)	107 (11; 189)	577 (392; 767)	1209 (981; 1431)	930 (697; 1154)	4157 (3508; 4826)	5183 (4366; 6043)	7339 (6187; 8476)

^a^Includes the following seasons: 2008/2009, 2010/2011, 2011/2012, 2012/2013, 2013/2014, 2014/2015, 2015/2016, 2016/2017, 2017/2018. Excludes season 2009/10, since it was affected by the 2009 influenza A (H1N1) pandemic

^b^Includes the most recent seasons, namely 2015/2016, 2016/2017, 2017/2018

**Table 5:** Estimated number of influenza-associated excess respiratory or cardiovascular hospitalizations per 100,000 people by age group and epidemic season in Portuguese public hospitals between 2008/2009 and 2017/2018

Season	Age groups (in years) 95% confidence interval							
	0–4	5–18	19–49	50–64	65–74	≥ 75	≥ 65	All ages
2008/ 2009	–	–	–	2.5 (− 7.1; 13.1)	83.4 (63.6; 101.7)	391.6 (332.2; 444.1)	229.7 (191.7; 261.6)	40.9 (30; 50.6)
2009/ 2010	18.3 (− 34.8; 73.2)	25.7 (20.8; 30.6)	36.5 (32.9; 40.4)	32.7 (23.6; 41.7)	–	–	–	16.4 (6.6; 25.6)
2010/ 2011	–	0.7 (− 5.7; 7.3)	21.8 (17; 26.8)	59 (48.5; 69)	63.4 (43.4; 83.6)	–	37.5 (2.2; 74.6)	26.7 (16.2; 37.5)
2011/ 2012	–	1 (− 4.6; 6.4)	9.1 (5.1; 13.6)	29.7 (20; 39.9)	112.6 (93.9; 130.3)	565.3 (511.9; 624.3)	336.9 (302.2; 371.8)	68.6 (58.4; 78.7)
2012/ 2013	–	–	5.8 (1.2; 10.5)	26.3 (15.4; 37.5)	31.8 (9.2; 54.4)	54.6 (− 11.6; 119.4)	50.4 (10.5; 92.2)	4.4 (− 7.7; 16)
2013/ 2014	83.4 (36.1; 134.3)	–	17.8 (13.9; 21.8)	45.5 (35.2; 55.6)	55.2 (36.9; 72.9)	108 (52; 160.5)	84.1 (50.2; 116.3)	32.5 (22.7; 42.2)
2014/ 2015	–	–	8.2 (4.7; 12)	41.7 (33.6; 51.5)	101.8 (85.7; 117.8)	595.8 (547.4; 645.9)	350.6 (322.6; 380.8)	77.1 (68.5; 86.3)
2015/ 2016	150.8 (87.2; 210.2)	18.9 (12.5; 24.9)	26.5 (21.7; 31.4)	62.8 (51.8; 73.9)	63.1 (41.2; 83.3)	216.2 (151.2; 282.6)	137.8 (98.6; 180)	56.6 (44.9; 68.3)
2016/ 2017	–	–	4.8 (0.6; 8.9)	42.5 (33.2; 51.5)	81.4 (64.8; 98.8)	585.9 (536.8; 639.2)	330.8 (300.6; 363.3)	85.2 (76.1; 94.4)
2017/ 2018	154.7 (85.1; 220.1)	3.1 (− 3.5; 10.1)	10.8 (6.2; 15.7)	64.9 (53.2; 76.1)	102.1 (78.6; 124.2)	365.7 (297.2; 434.2)	238.4 (196.1; 281.2)	71.9 (59.1; 84.1)
9 years mean^a^	43.2 (− 124.8; 122.2)	2.6 (− 6; 7.6)	11.6 (6.8; 15.7)	41.7 (31.5; 52)	77.2 (57.5; 96.3)	320.3 (261.9; 378.2)	199.6 (163.9; 235.8)	51.5 (40.9; 62)
3 years mean^b^	101.8 (27.2; 151.7)	7.3 (0.7; 12.9)	14 (9.5; 18.6)	56.7 (46.1; 67.2)	82.2 (61.5; 102.1)	389.3 (328.4; 452)	235.7 (198.5; 274.9)	71.2 (60.1; 82.3)

^a^Includes the following seasons: 2008/2009, 2010/2011, 2011/2012, 2012/2013, 2013/2014, 2014/2015, 2015/2016, 2016/2017, 2017/2018. Excludes season 2009/10, since it was affected by the 2009 influenza A (H1N1) pandemic

^b^Includes the most recent seasons, namely 2015/2016, 2016/2017, 2017/2018

### Influenza-associated excess mortality

We estimated a total of 21,334 (95% CI: 16,717; 27,332) influenza-associated excess all-cause deaths across the study period. Mean annual influenza-associated all-cause deaths per 100,000 people over nine seasons were estimated at 22.7 for all age groups^2^. Season 2014/15 was the most fatal, with 5,016 influenza-associated deaths (48.4 per 100,000). During the study period, influenza was accountable for deaths in all the analyzed age groups (Table 6). Influenza was associated with a particularly high annual excess number of all-cause deaths in people aged ≥ 65 years old in five seasons, namely 2008/09 (2966 deaths; 154.9 deaths per 100,000 people); 2011/12 (3634; 179.9), 2014/15 (4737; 223.0), 2016/17 (4111; 187.3), and 2017/18 (2651; 118.9). Absolute and relative estimated annual influenza-associated excess all-cause deaths per age group are detailed in Tables 6 and 7. Results for excess all-cause mortality are presented in this section, as they yielded the best model performance (Additional file 1: Table S3). Results for the other groups of causes are presented in the Additional file 1: Table S5.

**Table 6:** Estimated number of influenza-associated excess all-cause deaths by age group and epidemic season in Portuguese public hospitals between 2008/2009 and 2017/2018

Season	Age groups (in years) 95% confidence interval							
	0–4	5–18	19–49	50–64	65–74	≥ 75	≥ 65	All ages
2008/2009	9 (− 3; 20)	3 (− 6; 13)	112 (65; 159)	75 (2; 150)	374 (279; 462)	2571 (2099; 3062)	2966 (2433; 3513)	3301 (2661; 3921)
2009/2010	8 (− 4; 20)	–	72 (28; 118)	124 (56; 196)	28 (− 57; 119)	–	–	92 (− 516; 730)
2010/2011	7 (− 4; 18)	11 (3; 20)	161 (110; 215)	144 (64; 223)	211 (117; 307)	–	146 (− 489; 761)	451 (− 288; 1173)
2011/2012	21 (10; 32)	6 (− 2; 15)	–	173 (97; 255)	362 (261; 460)	3248 (2731; 3847)	3634 (3048; 4314)	3673 (3044; 4420)
2012/2013	8 (− 4; 21)	–	91 (37; 147)	186 (103; 282)	103 (− 9; 227)	925 (267; 1628)	1045 (314; 1848)	1188 (425; 2027)
2013/2014	9 (− 1; 19)	–	9 (− 35; 56)	54 (− 19; 133)	–	–	–	–
2014/2015	7 (− 1; 16)	–	52 (14; 89)	140 (70; 211)	399 (315; 493)	4342 (3858; 4863)	4737 (4200; 5295)	5016 (4444; 5648)
2015/2016	16 (4; 26)	–	–	93 (− 2; 181)	–	–	–	–
2016/2017	6 (− 2; 15)	6 (− 1; 14)	22 (− 20; 61)	282 (209; 359)	242 (145; 345)	3897 (3336; 4489)	4111 (3499; 4774)	4586 (3933; 5290)
2017/2018	3 (− 9; 16)	–	–	161 (61; 266)	272 (142; 408)	2293 (1575; 3109)	2651 (1834; 3556)	3028 (2208; 3990)
9-year mean^a^	10 (− 1; 20)	3 (− 9; 10)	50 (− 8; 101)	145 (65; 229)	218 (95; 352)	1919 (1125; 2694)	2143 (1259; 3008)	2345 (1468; 3325)
3-year mean^b^	8 (− 3; 19)	2 (− 11; 10)	7 (− 68; 51)	179 (89; 269)	171 (58; 290)	2063 (981; 3799)	2254 (1054; 4165)	2431 (1382; 4640)

^a^Includes the following seasons: 2008/2009, 2010/2011, 2011/2012, 2012/2013, 2013/2014, 2014/2015, 2015/2016, 2016/2017, 2017/2018. Excludes season 2009/10, since it was affected by the 2009 influenza A (H1N1) pandemic

^b^Includes the most recent seasons, namely 2015/2016, 2016/2017, 2017/2018

**Table 7:** Estimated number of influenza-associated excess all-cause deaths per 100,000 people by age group and epidemic season in Portuguese public hospitals between 2008/2009 and 2017/2018

Season	Age groups (in years) 95% confidence interval							
	0–4	5–18	19–49	50–64	65–74	≥ 75	≥ 65	All ages
2008/2009	1.8 (− 0.6;3.9)	0.2 (− 0.4;0.8)	2.4 (1.4;3.5)	3.8 (0.1;7.6)	36.9 (27.5;45.5)	285.9 (233.5;340.6)	154.9 (127.1;183.4)	31.2 (25.2;37.1)
2009/2010	1.5 (− 0.7;3.9)	–	1.6 (0.6;2.6)	6.2 (2.8;9.8)	2.8 (− 5.6;11.6)	–	–	0.9 (− 4.9;6.9)
2010/2011	1.4 (− 0.9;3.7)	0.7 (0.2;1.3)	3.6 (2.4;4.8)	7.1 (3.1;11)	20.5 (11.3;29.8)	–	7.3 (− 24.5;38.2)	4.3 (− 2.7;11.1)
2011/ 2012	4.3 (2.1;6.5)	0.4 (− 0.1;1)	–	8.4 (4.7;12.4)	35 (25.2;44.4)	329.8 (277.3;390.7)	179.9 (150.9;213.6)	34.9 (29;42)
2012/2013	1.8 (− 0.8;4.4)	–	2.1 (0.8;3.4)	9 (5;13.6)	9.8 (− 0.8;21.6)	92.1 (26.5;162.2)	50.9 (15.3;90.1)	11.4 (4.1;19.4)
2013/2014	2 (− 0.3;4.1)	–	0.2 (− 0.8;1.3)	2.6 (− 0.9;6.3)	–	–	–	–
2014/2015	1.7 (− 0.3;3.6)	–	1.2 (0.3;2.1)	6.7 (3.3;10)	36.9 (29.1;45.6)	417 (370.5;467)	223 (197.8;249.3)	48.4 (42.9;54.5)
2015/2016	3.6 (0.9;5.9)	–	–	4.4 (− 0.1;8.6)	–	–	–	–
2016/2017	1.5 (− 0.5;3.5)	0.4 (− 0.1;1)	0.5 (− 0.5;1.5)	13.2 (9.8;16.8)	21.4 (12.9;30.5)	365.6 (313;421.2)	187.3 (159.4;217.5)	44.5 (38.2;51.4)
2017/2018	0.7 (− 2.2;3.7)	–	–	7.5 (2.9;12.4)	23.7 (12.4;35.5)	212.3 (145.9;287.9)	118.9 (82.3;159.6)	29.4 (21.5;38.8)
9-year mean^a^	2.1 (− 0.3;4.4)	0.2 (− 0.6;0.7)	1.1 (− 0.2;2.3)	7 (3.1;11)	20.5 (9;32.9)	189.2 (111.3;265.8)	102.5 (60.4;144)	22.7 (14.1;32)
3-year mean^b^	1.9 (− 0.6;4.4)	0.1 (− 0.8;0.7)	0.2 (− 1.7;1.3)	8.4 (4.2;12.6)	15 (5;25.6)	192.6 (90.8;354.6)	102.1 (47;188.5)	24.6 (13.4;45.1)

^a^Includes the following seasons: 2008/2009, 2010/2011, 2011/2012, 2012/2013, 2013/2014, 2014/2015, 2015/2016, 2016/2017, 2017/2018. Excludes season 2009/10, since it was affected by the 2009 influenza A (H1N1) pandemic

^b^Includes the most recent seasons, namely 2015/2016, 2016/2017, 2017/2018

### Influenza economic burden

#### Hospitalizations coded as due to influenza

**Fig. 1:**
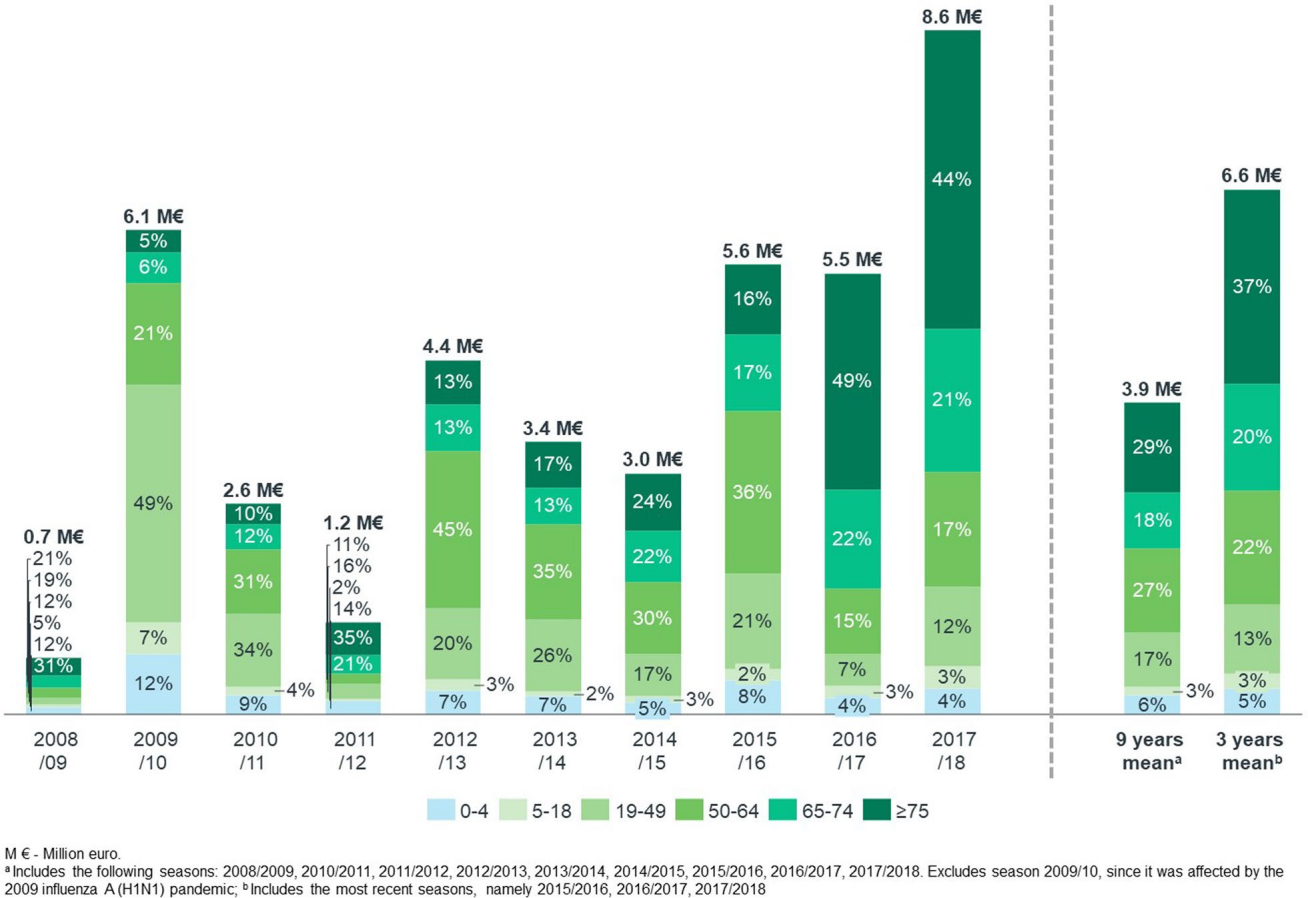
Direct cost of hospitalizations coded as due to influenza by age group and epidemic season in Portuguese public hospitals between 2008/2009 and 2017/2018 (million €)

Over ten years, the 13,629 influenza direct hospitalizations cost the Portuguese NHS €41.2 million. Costs varied considerably according to the epidemic season, from €0.7 million in 2008/09 to €8.6 million in 2017/18 (Fig. 1). The mean direct annual cost over nine seasons was €3.9 million^2^, 46.8% of which was generated by patients aged ≥ 65 years old, 44.2% by adult patients, and 9.0% by pediatric patients. Hospitalizations in which influenza was the primary diagnosis accounted for 53.7% of costs^2^. Patients with comorbidities, regardless of age, accounted for 78.6% of all influenza direct hospitalization costs^2^. At-risk patients, either because of their age (≥ 65 years) or due to underlying medical conditions, accounted for 88.4% of all influenza hospitalization costs^2^. Table 3 presents the mean influenza direct hospitalization cost per patient, according to age and the comorbidity profile.

### Influenza-associated excess respiratory or cardiovascular hospitalization

**Fig. 2:**
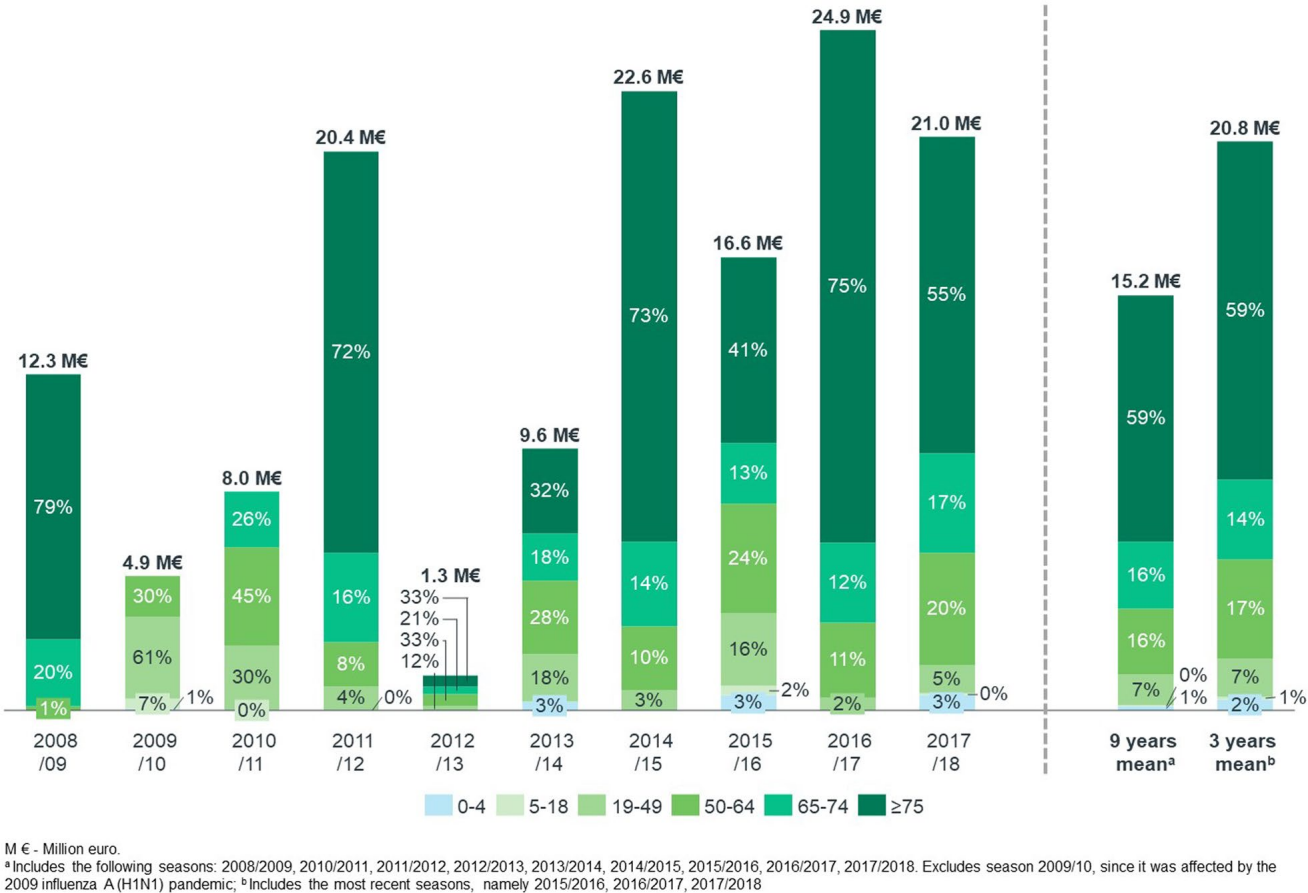
Direct cost of influenza-associated excess respiratory or cardiovascular hospitalizations by age group and epidemic season in Portuguese public hospitals between 2008/2009 and 2017/2018 (million €). The total cost corresponds to the cost of all-age influenza-associated excess R&C hospitalization. The percentage for each age group was computed as a proportion of the sum of influenza-associated excess R&C hospitalization estimated for each individual age group

Over ten years, the direct cost of the 49,929 all-age influenza-associated excess R&C hospitalizations was estimated at €141.5 million. The population aged ≥ 65 years old was associated with the highest total hospitalization cost: the 37,737 influenza-associated excess R&C hospitalizations were estimated to have cost the NHS €114.9 million. The mean direct annual^2^ cost of influenza-associated excess R&C hospitalizations was €15.2 million for all age groups and €12.8 million for the population aged ≥ 65 years old (both excluding the 2009/10 pandemic season) (Fig. 2).

## Discussion

This is the first national study to estimate the global epidemiologic and economic morbidity and mortality burden of influenza by integrating two complementary approaches: cases actually coded with influenza-specific diagnosis and cases estimated to be due to influenza based on statistical excess modeling techniques—in an attempt to capture influenza-related complications. The results were assessed across ten epidemic seasons, six age groups, and four groups of diagnoses. The mortality data are representative of the country, as all deaths registered in the nation were used. As for hospitalization data, they represent all NHS hospitalizations, not including the private sector. The results provide critical information for policymakers.

Firstly, the findings confirm the high burden of severe influenza in Portugal, both in terms of estimated influenza-associated excess R&C hospitalizations and all-cause deaths. We estimate that influenza was associated with five thousand (5,016) deaths during the most severe season (2014/2015), which is consistent with estimates from the INSA (4961 all-cause total deaths) [6]. In the last analyzed season (2017/18), we estimated the overall influenza-associated all-cause mortality to be 29.4 (95% CI 21.5–38.8) per 100,000 population and 118.9 (95% CI 82.3–159.6) in those aged ≥ 65 years, which is similar to the mean rates estimated by Nielsen et al. for 24 European countries, namely 25.4 and 118.2 for all age groups and those aged ≥ 65 years old, respectively [22].

The results confirm the relevance of analyzing excess morbidity and mortality in each season segregated by age and by groups of causes, as relevant variations are observed across time. From our study, we cannot ascertain to what degree this is due to the severity of different strains or the level of mismatch with vaccine strains.

Importantly, the study demonstrates how the burden of severe influenza may be substantially underestimated if only administrative data with influenza-specific codes are used. Excluding the 2009/2010 pandemic season, hospitalizations coded as due to influenza represented only 22.5% of the estimated influenza-associated excess R&C hospitalization in all age groups and 13.3% in the population aged ≥ 65 years old, corresponding to a ratio of 5.1 and 11.2 influenza-associated R&C hospitalizations for every hospitalization actually coded with influenza-specific codes^2^. As for deaths, those observed during hospitalizations coded as due to influenza represented only 3.1% and 2.5% of the estimated influenza-associated excess all-cause deaths, in all age groups and in the population aged ≥ 65 years old, respectively^2^. If only hospitalizations in which influenza was registered as a primary diagnosis were considered, they would represent only 1.5% of all-age and all-cause estimated influenza-associated excess deaths and 14.7% of influenza-associated R&C excess hospitalizations^2^. A study performed in Colorado, USA, estimated four influenza-associated excess R&C hospitalizations for each influenza-coded hospitalization from hospital discharge records in the population aged ≥ 65 years old. The difference was higher for mortality, with a ratio of 10.1 R&C deaths estimated to be influenza-associated per death, with influenza listed as the cause on the death certificate [15]. Both ratios were lower in the population aged < 65 years old, which is consistent with our findings. However, we observe an apparent higher under-detection in Portugal across all age groups. A notable exception was the 2009/2010 influenza H1N1 pandemic, in which all-cause deaths observed during hospitalizations coded as due to influenza represented 72.6% of the estimated influenza-associated excess all-cause deaths in that season, suggesting greater testing for influenza during pandemics, or a reflex of the reduced severity of the pandemic H1N1 virus, leading to fewer deaths than usually observed during influenza seasons. The under-detection factor was smaller in the last analyzed season, but is still not sufficient to capture a great part of the burden of severe influenza. The presented evidence signals the need for increased testing for influenza, as it can trigger various complications that can lead to hospitalizations and deaths [2], as well as the importance of considering its under-detection when assessing the clinical and economic burden, as performed in other geographies such as the USA [23]. It also signals the importance of having data on weekly ILI incidence rates per age group to enable different circulations of the virus, thus potentially improving the performance of models for younger age groups [24].

As expected, most hospitalizations and deaths were observed in elderly patients, who also had the worst outcomes, resulting in higher resource consumption. However, relevant excess hospitalizations and mortality were also observed in younger age groups. Concretely, two age groups merit further study and reflection regarding proper preventive measures, namely those aged 50–64 years old and those aged < 5 years old. The first group was associated with the highest mean LoS (13.2 days^2^) and use of invasive mechanical ventilation (in 17.4% of hospitalizations^2^), which suggests that a relevant burden can also be expected with patients’ recovery after discharge. As for the younger age group, data suggest a relevant number of annual excess R&C hospitalizations and all-cause deaths in some seasons. Populations with comorbidities, regardless of age, had the longest hospital stays, a higher in-hospital case fatality risk, and higher use of invasive and non-invasive mechanical ventilation.

The results reported in this study should be interpreted in light of several limitations. Regarding the analysis on hospitalizations coded as due to influenza, this entail the usual limitations related to a study based on administrative data (e.g., no laboratory data, no data on whether the subjects had been vaccinated, subject to coding errors or missing information). Furthermore, the study period included two ICD systems, with ICD10 offering greater granularity, which might have impacted coding practices. The excess modeling analysis was used to help overcome a few of these limitations. As for the excess hospitalization and mortality models, we used ILI incidence as an indicator of influenza activity, without further control variables, based on published literature [13, 15]. Other variables could have also been used, such as laboratory data on the circulation of other respiratory viruses [25, 26]. In France, a comparison of models using ILI and percent influenza positive demonstrated that ILI was the most statistically relevant indicator of mortality and that, most importantly, both indicators produced similar influenza burden estimates [13]. Nonetheless, these findings may not necessarily hold in Portugal. The choice to use ILI indicator for influenza activity in the model may be a limitation of our work, as ILI may capture diseases caused by other respiratory pathogens. Hence excess mortality modeled with ILI cannot strictly be attributed to influenza alone. Still, the models employed in the BARI study presented global high performance in terms of correlation and MAPE (for R&C hospitalizations, the correlation was between 91 and 97% and for MAPE, between 3 and 10%, depending on the age group, as presented in the Supplementary Materials). The age groups used in our study were defined by a panel of experts (co-authors), considering their relevance at the national level for the elaboration of vaccination policies. However, they do not follow the WHO standard age groupings for influenza surveillance [27], which, if used, would facilitate comparisons with estimates from other countries, as well as potential meta-analyses in the future.

The study estimated a mean annual direct cost of influenza-associated excess R&C hospitalizations of €15.2 million, which is 3.9 higher than the estimates obtained by considering only the costs of hospitalizations coded as due to influenza^2^. However, despite providing a broader perspective on the burden of severe influenza, the study still does not consider the full burden of influenza. Hospitalizations in the private setting, which represented 22.9% of total hospitalizations in Portugal in 2018 [28], were not included. All things being equal between public and private hospitals, excluding private hospitals from the analysis could underestimate the influenza burden by ~ 20%, which is substantial. The burden of milder influenza cases with self-management or managed in a primary health care setting was also not accounted for [10]. Indirect costs of lost productivity from patients (or their parents) were not computed, nor were the medium and long-term costs related to the posterior treatment of complications caused or aggravated by influenza.

Generating more comprehensive evidence contributes to a better understanding of the implications that influenza can have for public health and may also help generate awareness and strengthen preventive policies. Further studies analyzing the impact of influenza in relation to specific complications could enable more targeted interventions, a better understanding of the long-term impact of severe illness on survivors, and other non-hospitalization related costs.

## Conclusions

Influenza is a respiratory disease that burdens the health care system every winter. In our study, we observed a strong burden of influenza and its complications on mortality and health care resource utilization at the hospital level, which is not always captured by routine surveillance systems. We conclude that there is still a substantial burden of influenza in not only the elderly but also the younger population that remains to be addressed. Vaccination is a safe and effective preventive measure that could help preserve the health of at-risk individuals, decrease the social and economic impact of influenza and contribute to the sustainability of health systems.

## Supplementary Information

**Additional file 1: Table S1**. Diagnostic codes used to identify comorbidities/risk factors for influenza: a) in people ≥5 years old; b) inchildren <5 years old. **Table S2**. Performance of the excess hospitalization model per age group and cause. **Table S3**. Performance of the excess deaths model per age group and cause. **Table S4**. Estimated number of influenza-associated excess pneumonia or influenza, respiratory, and all-cause hospitalization by age group and epidemic season in Portuguese public hospitals between 2008/2009 and 2017/2018. **Table S5**. Estimated number of influenza-associated excess pneumonia or influenza, respiratory, and respiratory or cardiovascular deaths by age group and epidemic season in Portuguese public hospitals between 2008/2009 and 2017/2018. **Table S6**. Ninety-five percent confidence intervals of estimated number of influenza-associated excess pneumonia or influenza, respiratory, and all-cause hospitalization by age group and epidemic season in Portuguese public hospitals between 2008/2009 and 2017/2018. **Table S7**. Ninety-five percent confidence intervals of estimated number of influenza-associated excess pneumonia or influenza, respiratory, and respiratory or cardiovascular deaths by age group and epidemic season in Portuguese public hospitals between 2008/2009 and 2017/2018.

## Data Availability

The data that support the study's findings are available from IQVIA, but there are restrictions on their availability because they were used under license for the current study and thus are not publicly available. Data are, however, available from the authors upon reasonable request and with permission from IQVIA.

## Abbreviations

AEMPS: Agency of medicines and medical devices
BARI: Burden of acute respiratory infections
CI: Confidence interval
DRG: Diagnosis-related group
ICD: International classification of diseases
ILI: Influenza-like illness
INE: Instituto Nacional de Estatística
INSA: Instituto Nacional de Saúde Doutor Ricardo Jorge
LoS: Length of stay
MAPE: Mean absolute percentage error
NHS: National Health Service
P&I: Pneumonia and/or influenza
R&C: Respiratory and/or cardiovascular
WHO: World Health Organization

## Footnotes

1 As for the European Union, the recommendation on annual vaccination against influenza in children is targeted to those with either weakened immune systems or chronic health conditions.

2 Mean for nine seasons (2008/2009 to 2017/2018), ie, excluding the H1N1pdm09 pandemic (2009/10).
