# Supplementary material for: Excess hospitalizations and mortality associated with seasonal influenza in Portugal, 2008–2018
Source: BMC Infect Dis. 2022 Sep 7;22:726. doi: 10.1186/s12879-022-07713-8 (PMC9450401; doi:10.1186/s12879-022-07713-8)
Supplement: Supplementary file 1 — Additional file 1: Table S1. Diagnostic codes used to identify comorbidities/risk factors for influenza: a) in people ≥5 years old; b) inchildren <5 years old. Table S2. Performance of the excess hospitalization model per age group and cause. Table S3. Performance of the excess deaths model per age group and cause. Table S4. Estimated number of influenza-associated excess pneumonia or influenza, respiratory, and all-cause hospitalization by age group and epidemic season in Portuguese public hospitals between 2008/2009 and 2017/2018. Table S5. Estimated number of influenza-associated excess pneumonia or influenza, respiratory, and respiratory or cardiovascular deaths by age group and epidemic season in Portuguese public hospitals between 2008/2009 and 2017/2018. Table S6. Ninety-five percent confidence intervals of estimated number of influenza-associated excess pneumonia or influenza, respiratory, and all-cause hospitalization by age group and epidemic season in Portuguese public hospitals between 2008/2009 and 2017/2018. Table S7. Ninety-five percent confidence intervals of estimated number of influenza-associated excess pneumonia or influenza, respiratory, and respiratory or cardiovascular deaths by age group and epidemic season in Portuguese public hospitals between 2008/2009 and 2017/2018. [file 12879_2022_7713_MOESM1_ESM.docx]

### Table S1. Diagnostic codes used to identify comorbidities/risk factors for influenza: a) in people ≥5 years old; b) in children <5 years old

1. Diagnostic codes used to identify comorbidities/risk factors for influenza in people ≥5 years old

| Broader | Narrow | ICD-9 Diagnosis codes | ICD-10 Diagnosis Codes |
| --- | --- | --- | --- |
| Pregnancy | | 633.00 – 633.91; V22.0 – V23.9 | Z33 – Z34.93 |
| Diabetes Mellitus | | 250.xx | E08- E13 |
| Respiratory/  lung | Bronchiectasis | 748.61, 494.0, 494.1, 011.5 | J47.0, J47.1, J47.9, Q33.4 |
|  | Asthma | 493.xx | J45.xx (J45.20, J45.21, J45.22, J45.30, J45.31, J45.32, J45.40, J45.41, J45.42, J45.50, J45.51, J45.52, J45.901, J45.902, J45.909, J45.991, J45.998) |
|  | Chronic obstructive pulmonary disease (COPD) | 490-492.x; 496 | J40, J41.0, J41.1, J41.8, J42, J43.0, J43.1, J43.2, J43.8, J43.9, J44.0, J44.1, J44.9 |
|  | Post inflammatory pulmonary fibrosis, Chronic and other pulmonary manifestations due to radiation, Chronic respiratory disease arising in the perinatal period, Idiopathic fibrosing alveolitis, With pulmonary manifestations | 515, 508.1, 770.7, 516.3, 277.02 | J84.10, J70.1, P27, J84.112, E84.0 |
|  | Rheumatoid lung | 714.81 | M05.1 |
| Cardiovascular | Chronic cardiac disease | 393.xx – 398, 410.xx – 417.xx; 420.xx – 429.xx | I05.xx – I09.xx, I20.xx – I25.xx; I26.xx – 128.xx; I30.xx – I.52.xx; T80.0; T81.71; T81.72; T82.81 |
|  | Hypertensive Disease | 401-40 | I11.0, I11.9, I16.0, I16.1, I16.9 |
|  | CVD | 430 - 438.xx | I60-I69; G45-G46; |
|  | Peripheral vascular disease | 443.9 | I73.9 |
|  | High risk congenital heart disease | 745.0 - 745.4; 745.6x - 745.8; 746.01 - 746.5; 746.7 - 746.85; 746.87; 747.1x; 747.21 -747.49 | Q20-Q26 |
|  | Low risk congenital heart disease | 745.5; 745.9; 746.00; 746.6; 746.86; 746.89; 746.9; 747.0; 747.20; 747.83 | P29.3; Q20.9; Q21.1; Q21.9; Q22.3; Q23.3;  Q23.8; Q23.9; Q24.6; Q24.8; Q24.9; Q25.0; Q25.4 |
| Immuno-compromised | HSCT | 996.88, 996.85, 41.04 - 41.09 | T86.5. |
|  | Lung transplant | 996.84; V42.6, 32.3x - 32.5x; 33.5x | Z94.2, T86.83  T86.81, |
|  | HIV | 042.xx | B20 |
|  | Hematologic malignancy (leukemia, lymphoma, multiple myeloma) | 203.xx - 208.xx; 238.4; 238.72 - 238.76; 289.83 | C81-C96 |
|  | Non-lung solid organ transplant | 199.2; 996.52; 996.55; 996.80 - 996.83; 996.86 - 996.89; E878.0; V42.0 - V42.3; V42.7; V42.83; V42.84; V45.87; V58.44, 07.94; 37.51; 41.94; 46.97; 50.51; 50.59; 52.80; 52.82; 52.83; 55.53; 55.69 | Z94.0, Z94.1, Z94.4, Z94.5, Z94.7, T86.1, T86.2, T86.4, Z98.85, Z48.288, Z48.298, |
|  | Hereditary haemolytic anaemias | 282.xx | D55.xx – D59.xx |
|  | Other immune deficiencies | 279.xx | D80.xx – D89.xx |
| Chronic liver disease |  | 571.xx | K70.xx – K77.xx |
| Chronic kidney disease |  | 403.00, 403.1, 585.1, 585.2, 585.3, 585.4, 585.5, 585.6, 585.9 | I12.0, I12.9, N18.1, N18.2, N18.3, N18.4, N18.5, N18.6, N18. |

1. Diagnostic codes used to identify comorbidities/risk factors for influenza in children <5 years old

| Broader | Narrow | ICD-9 Diagnosis codes | ICD-10 |
| --- | --- | --- | --- |
| Cardiopulmonary | Chronic cardiac disease | 393.xx – 398, 410.xx – 417.xx; 420.xx – 429.xx | I05.xx – I09.xx, I20.xx – I25.xx; I26.xx – 128.xx; I30.xx – I.52.xx; T80.0; T81.71; T81.72; T82.81 |
|  | CVD | 430 - 438.xx | I60-I69; G45-G46; |
|  | Hypertension | 401.xx – 405.xx | I11.0, I11.9, I16.0, I16.1, I16.9 |
|  | Peripheral vascular disease | 443.9 | I73.9 |
|  | High risk congenital heart disease | 745.0 - 745.4; 745.6x - 745.8; 746.01 - 746.5; 746.7 - 746.85; 746.87; 747.1x; 747.21 - 747.49 | Q20-Q26 |
|  | Low risk congenital heart disease | 745.5; 745.9; 746.00; 746.6; 746.86; 746.89; 746.9; 747.0; 747.20; 747.83 | P29.3; Q20.9; Q21.1; Q21.9; Q22.3; Q23.3;  Q23.8; Q23.9; Q24.6; Q24.8; Q24.9; Q25.0; Q25.4 |
|  | BDP | 770.7x | P27 |
| Respiratory/Lung disease | Bronchiectasis | 494, 748.61, 494.0, 011.5 | J47.0, J47.1, J47.9, Q33.4 |
|  | Interstitial pulmonary fibrosis of prematurity, Wilson-Mikity syndrome | 770.7x | P 27 |
|  | Wilson-Mikity syndrome | 770.7x | P27.0 |
|  | Congenital anomalies of respiratory system | 748., | Q30-Q34 |
|  | Chronic perinatal respiratory disease | 770.7x | P28 |
|  | Other lung pathologies | 507.1; 514; 516.8; 517.2 - 517.8 | J69, J84.09, M34.8 |
|  | Cystic fibrosis | 277.0x | E84 |
| Other comorbidities | Down syndrome with CHD | 758.0 (down syndrome) | Q90 |
|  | Neuromuscular impairment | 330.x; 335.xx; 343.x; 356.x; 358.1; 359.0 - 359.23 | E75.0; E75.1; E75.2; E75.4; F84.2; G12; G31.8; G31.9; G60; G71.0-G71.3; G80 (excl. G80.3); G93.8; G93.9 |
| Immunocompromised | HSCT | 996.88, 996.85, 41.04 - 41.09 | T86.5. |
|  | Lung transplant | 996.84; V42.6, 32.3x - 32.5x; 33.5x | Z94.2, T86.83  T86.81, |
|  | HIV | 042.xx | B20 |
|  | Hematologic malignancy (leukemia, lymphoma, multiple myeloma) | 203.xx - 208.xx; 238.4; 238.72 - 238.76; 289.83 | C81-C96 |
|  | Non-lung solid organ transplant | 199.2; 996.52; 996.55; 996.80 - 996.83; 996.86 - 996.89; E878.0; V42.0 - V42.3; V42.7; V42.83; V42.84; V45.87; V58.44, 07.94; 37.51; 41.94; 46.97; 50.51; 50.59; 52.80; 52.82; 52.83; 55.53; 55.69 | Z94.0, Z94.1, Z94.4, Z94.5, Z94.7, T86.1, T86.2, T86.4, Z98.85, Z48.288, Z48.298, |
|  | Hereditary haemolytic anaemias | 282.xx | D55.xx – D59.xx |
|  | Other immune deficiencies | 279.xx | D80.xx – D89.xx |

### Table S2. Performance of the excess hospitalization model per age group and cause

| Age groups (in years) | P&I | | Respiratory | | Cardio-respiratory | | All-cause | |
| --- | --- | --- | --- | --- | --- | --- | --- | --- |
|  | r | MAPE | r | MAPE | r | MAPE | r | MAPE |
| 0-4 | 96% | 16% | 97% | 10% | 97% | 10% | 85% | 3% |
| 5-18 | 90% | 20% | 91% | 7% | 91% | 7% | 88% | 4% |
| 19-49 | 93% | 13% | 91% | 6% | 93% | 4% | 91% | 3% |
| 50-64 | 96% | 8% | 97% | 5% | 96% | 3% | 92% | 3% |
| 65-74 | 96% | 7% | 97% | 5% | 96% | 3% | 89% | 3% |
| ≥75 | 98% | 5% | 98% | 4% | 97% | 3% | 91% | 3% |
| ≥65 | 98% | 5% | 98% | 4% | 97% | 3% | 89% | 2% |
| All ages | 98% | 6% | 98% | 5% | 98% | 3% | 93% | 2% |

r – Person’s correlation; MAPE; Mean absolute percentage error.

### Table S3. Performance of the excess deaths model per age group and cause

| Age groups (in years) | P&I | | Respiratory | | Cardio-respiratory | | All-cause | |
| --- | --- | --- | --- | --- | --- | --- | --- | --- |
|  | R | MAPE | r | MAPE | r | MAPE | r | MAPE |
| 0-4 |  |  | 64% | 53% | 50% | 45% | 41% | 21% |
| 5-18 | 45% | 72% | 51% | 54% | 40% | 34% | 60% | 22% |
| 19-49 | 71% | 48% | 79% | 41% | 73% | 14% | 90% | 5% |
| 50-64 | 80% | 28% | 87% | 17% | 87% | 8% | 87% | 4% |
| 65-74 | 77% | 22% | 90% | 12% | 95% | 5% | 95% | 3% |
| ≥75 | 87% | 13% | 95% | 7% | 96% | 4% | 97% | 3% |
| ≥65 | 87% | 13% | 95% | 7% | 96% | 4% | 97% | 3% |
| All ages | 94% | 10% | 95% | 7% | 96% | 4% | 96% | 3% |

r – Person’s correlation; MAPE - Mean absolute percentage error.

### Table S4. Estimated number of influenza-associated excess pneumonia or influenza, respiratory, and all-cause hospitalization by age group and epidemic season in Portuguese public hospitals between 2008/2009 and 2017/2018

| Season | Age groups (in years) | | | | | | | |
| --- | --- | --- | --- | --- | --- | --- | --- | --- |
|  | 0-4 | 5-18 | 19-49 | 50-64 | 65-74 | ≥75 | ≥65 | All ages |
| P&I influenza-associated hospitalizations | | | | | | | | |
| 2008/2009 | 119 | 25 | - | 106 | 243 | 1,579 | 1,850 | 2,483 |
| 2009/2010 | 570 | 495 | 1,513 | 724 | 209 | - | 85 | 3,659 |
| 2010/2011 | 183 | 90 | 705 | 576 | 388 | 435 | 834 | 2,749 |
| 2011/2012 | 40 | 59 | 118 | 315 | 721 | 3,030 | 3,731 | 4,280 |
| 2012/2013 | - | - | 263 | 307 | 149 | 331 | 477 | 947 |
| 2013/2014 | 259 | 77 | 454 | 515 | 354 | 331 | 648 | 2,069 |
| 2014/2015 | 84 | 118 | 317 | 512 | 652 | 3,195 | 3,831 | 5,083 |
| 2015/2016 | 278 | 97 | 555 | 695 | 568 | 950 | 1,456 | 3,172 |
| 2016/2017 | 101 | 50 | 196 | 535 | 723 | 3,789 | 4,510 | 5,569 |
| 2017/2018 | 215 | 146 | 367 | 701 | 794 | 2,748 | 3,558 | 4,948 |
| Respiratory influenza-associated hospitalizations | | | | | | | | |
| 2008/2009 | - | - | - | 247 | 713 | 2,834 | 3,514 | 3,407 |
| 2009/2010 | 95 | 430 | 1,713 | 779 | 170 | - | - | 2,779 |
| 2010/2011 | - | 18 | 844 | 1,009 | 624 | 510 | 1,108 | 3,112 |
| 2011/2012 | - | 23 | 400 | 630 | 1,190 | 5,210 | 6,412 | 7,282 |
| 2012/2013 | - | - | 252 | 572 | 351 | 770 | 1,099 | 1,210 |
| 2013/2014 | 367 | - | 763 | 829 | 563 | 1,068 | 1,591 | 3,528 |
| 2014/2015 | - | 17 | 284 | 792 | 1,116 | 5,100 | 6,162 | 6,923 |
| 2015/2016 | 646 | 263 | 886 | 957 | 570 | 1,396 | 1,853 | 4,477 |
| 2016/2017 | - | - | 109 | 850 | 1,151 | 5,690 | 6,782 | 8,122 |
| 2017/2018 | 652 | 47 | 543 | 1,201 | 1,271 | 4,245 | 5,475 | 7,479 |
| All-cause influenza-associated hospitalizations | | | | | | | | |
| 2008/2009 | - | - | - | - | 72 | 3,262 | 2,690 | - |
| 2009/2010 | - | - | 409 | 622 | - | - | - | - |
| 2010/2011 | 922 | - | 2,843 | 1,596 | 724 | - | - | 6,316 |
| 2011/2012 | 214 | - | - | 742 | 1,788 | 5,385 | 7,193 | 6,781 |
| 2012/2013 | - | - | - | 797 | 19 | - | - | - |
| 2013/2014 | - | 211 | - | 1,788 | 1,004 | 1,570 | 2,586 | 3,148 |
| 2014/2015 | - | 160 | - | 698 | 592 | 6,476 | 6,670 | 6,087 |
| 2015/2016 | 743 | 667 | 1,252 | 2,735 | 1,322 | 3,343 | 4,533 | 10,205 |
| 2016/2017 | - | - | - | 709 | 427 | 6,254 | 6,205 | 6,463 |
| 2017/2018 | - | 101 | - | 2,192 | 978 | 2,817 | 3,938 | 7,203 |

### Table S5. Estimated number of influenza-associated excess pneumonia or influenza, respiratory, and respiratory or cardiovascular deaths by age group and epidemic season in Portuguese public hospitals between 2008/2009 and 2017/2018

| Season | Age groups (in years) | | | | | | | |
| --- | --- | --- | --- | --- | --- | --- | --- | --- |
|  | 0-4 | 5-18 | 19-49 | 50-64 | 65-74 | ≥75 | ≥65 | All ages |
| P&I influenza-associated deaths | | | | | | | | |
| 2008/2009 | -^a^ | 0 | 7 | 10 | 12 | 407 | 421 | 435 |
| 2009/2010 |  | - | 25 | - | - | - | - | - |
| 2010/2011 |  | 1 | 7 | 6 | 2 | - | - | - |
| 2011/2012 |  | 1 | 11 | 0 | 77 | 882 | 963 | 960 |
| 2012/2013 |  | 1 | 14 | 6 | 19 | 227 | 244 | 267 |
| 2013/2014 |  | 0 | 16 | 1 | - | 50 | 40 | 61 |
| 2014/2015 |  | 0 | 13 | 44 | 56 | 807 | 858 | 920 |
| 2015/2016 |  | 5 | 29 | 49 | 26 | 115 | 130 | 210 |
| 2016/2017 |  | 2 | 1 | 31 | 66 | 658 | 715 | 736 |
| 2017/2018 |  | 1 | 22 | 50 | 54 | 701 | 762 | 814 |
| Respiratory influenza-associated deaths | | | | | | | | |
| 2008/2009 | 4 | 1 | 29 | 19 | 80 | 848 | 900 | 909 |
| 2009/2010 | 0 | 3 | 68 | 32 | 19 | - | - | - |
| 2010/2011 | 5 | 5 | 44 | 81 | 61 | 76 | 131 | 257 |
| 2011/2012 | - | 5 | 18 | 8 | 141 | 1,472 | 1,637 | 1,670 |
| 2012/2013 | - | 2 | 28 | 39 | 1 | 262 | 276 | 369 |
| 2013/2014 | 0 | 1 | 29 | 32 | - | 2 | - | 80 |
| 2014/2015 | - | 2 | 19 | 83 | 140 | 1,533 | 1,652 | 1,767 |
| 2015/2016 | - | 5 | 43 | 64 | 46 | 44 | 50 | 171 |
| 2016/2017 | - | 0 | 15 | 45 | 108 | 1,207 | 1,272 | 1,298 |
| 2017/2018 | 0 | - | 25 | 59 | 133 | 1,121 | 1,240 | 1,260 |
| R&C influenza-associated deaths | | | | | | | | |
| 2008/2009 | 4 | 2 | 30 | 80 | 229 | 1,870 | 2,118 | 2,297 |
| 2009/2010 | 0 | 4 | 85 | 48 | 1 | - | - | - |
| 2010/2011 | 5 | 7 | 45 | 120 | 81 | 89 | 221 | 460 |
| 2011/2012 | - | 3 | 19 | - | 127 | 2,180 | 2,319 | 2,253 |
| 2012/2013 | - | 1 | - | 7 | - | 498 | 423 | 460 |
| 2013/2014 | 1 | 2 | 73 | 16 | - | - | - | - |
| 2014/2015 | 1 | 3 | 34 | 153 | 277 | 3,009 | 3,266 | 3,523 |
| 2015/2016 | 0 | 5 | 54 | 99 | 97 | - | - | - |
| 2016/2017 | - | 3 | 16 | 111 | 193 | 2,567 | 2,729 | 2,898 |
| 2017/2018 | 1 | - | - | 22 | 298 | 1,439 | 1,758 | 1,685 |

a. Not estimated due to the low number of deaths in this age group.

### Table S6. Ninety-five percent confidence intervals of estimated number of influenza-associated excess pneumonia or influenza, respiratory, and all-cause hospitalization by age group and epidemic season in Portuguese public hospitals between 2008/2009 and 2017/2018

| Season | Age groups (in years)  95% confidence interval | | | | | | | |
| --- | --- | --- | --- | --- | --- | --- | --- | --- |
|  | 0-4 | 5-18 | 19-49 | 50-64 | 65-74 | ≥75 | ≥65 | All ages |
| P&I influenza-associated hospitalizations | | | | | | | | |
| 2008/2009 | (40; 207) | (-19; 66) | (-87; 76) | (43; 166) | (176; 307) | (1375; 1758) | (1602; 2073) | (2110; 2851) |
| 2009/2010 | (512; 629) | (458; 516) | (1439; 1551) | (665; 785) | (145; 273) | (-308; 85) | (-157; 334) | (3312; 4037) |
| 2010/2011 | (115; 262) | (49; 133) | (644; 776) | (510; 648) | (320; 457) | (217; 670) | (566; 1123) | (2350; 3177) |
| 2011/2012 | (-28; 103) | (25; 92) | (54; 189) | (247; 386) | (658; 783) | (2828; 3242) | (3480; 3991) | (3920; 4660) |
| 2012/2013 | (-266; -119) | (-146; -63) | (194; 340) | (236; 390) | (70; 232) | (79; 595) | (172; 799) | (515; 1399) |
| 2013/2014 | (198; 312) | (45; 111) | (399; 515) | (451; 584) | (288; 418) | (91; 566) | (362; 934) | (1682; 2458) |
| 2014/2015 | (27; 135) | (87; 146) | (266; 371) | (455; 574) | (593; 715) | (2987; 3393) | (3576; 4072) | (4736; 5430) |
| 2015/2016 | (207; 334) | (56; 137) | (491; 623) | (615; 769) | (479; 651) | (673; 1219) | (1111; 1786) | (2712; 3621) |
| 2016/2017 | (40; 155) | (14; 81) | (133; 258) | (473; 604) | (659; 787) | (3594; 4014) | (4268; 4785) | (5232; 5930) |
| 2017/2018 | (132; 281) | (111; 182) | (299; 448) | (618; 778) | (710; 875) | (2446; 3041) | (3185; 3922) | (4453; 5418) |
| Respiratory influenza-associated hospitalizations | | | | | | | | |
| 2008/2009 | (-1313; -658) | (-144; 37) | (-276; 30) | (148; 345) | (600; 808) | (2507; 3121) | (3097; 3888) | (2771; 4048) |
| 2009/2010 | (-171; 374) | (353; 503) | (1596; 1832) | (688; 876) | (66; 277) | (-662; 1) | (-613; 213) | (2148; 3418) |
| 2010/2011 | (-1147; -533) | (-76; 115) | (696; 1001) | (905; 1119) | (510; 737) | (154; 879) | (667; 1560) | (2383; 3847) |
| 2011/2012 | (-412; 99) | (-62; 103) | (272; 545) | (529; 733) | (1088; 1293) | (4873; 5594) | (6001; 6873) | (6637; 7981) |
| 2012/2013 | (-1740; -1158) | (-319; -123) | (101; 405) | (466; 691) | (226; 479) | (337; 1204) | (564; 1620) | (427; 1936) |
| 2013/2014 | (152; 596) | (-130; 34) | (645; 885) | (722; 931) | (447; 665) | (685; 1427) | (1106; 2039) | (2862; 4178) |
| 2014/2015 | (-1287; -817) | (-54; 85) | (176; 402) | (700; 893) | (1017; 1224) | (4747; 5420) | (5730; 6568) | (6301; 7542) |
| 2015/2016 | (373; 895) | (173; 348) | (739; 1029) | (825; 1075) | (431; 701) | (932; 1849) | (1291; 2441) | (3649; 5260) |
| 2016/2017 | (-382; 109) | (-98; 53) | (-15; 234) | (756; 956) | (1042; 1261) | (5364; 6081) | (6378; 7263) | (7496; 8810) |
| 2017/2018 | (351; 929) | (-46; 146) | (411; 688) | (1068; 1327) | (1134; 1400) | (3741; 4724) | (4858; 6074) | (6629; 8322) |
| All-cause influenza-associated hospitalizations | | | | | | | | |
| 2008/2009 | (-2442; -1097) | (-665; -117) | (-4686; -2345) | (-1029; 640) | (-663; 731) | (2340; 4141) | (1167; 4123) | (-5323; 2806) |
| 2009/2010 | (-994; 145) | (-357; 155) | (-652; 1419) | (-167; 1412) | (-1206; 23) | (-1852; -4) | (-3277; -394) | (-5505; 1939) |
| 2010/2011 | (323; 1576) | (-336; 208) | (1754; 3884) | (731; 2443) | (3; 1425) | (-1282; 872) | (-1696; 1466) | (2130; 10438) |
| 2011/2012 | (-428; 862) | (-422; 96) | (-1805; 246) | (-138; 1650) | (1105; 2504) | (4392; 6397) | (5583; 8732) | (2864; 10547) |
| 2012/2013 | (-4506; -3112) | (-758; -193) | (-3977; -1652) | (-198; 1696) | (-823; 812) | (-1306; 1210) | (-2421; 1598) | (-10429; -1212) |
| 2013/2014 | (-1718; -516) | (-30; 452) | (-1236; 709) | (929; 2629) | (372; 1686) | (529; 2607) | (1019; 4115) | (-594; 6851) |
| 2014/2015 | (-1934; -928) | (-60; 380) | (-2073; -417) | (-83; 1464) | (-20; 1229) | (5548; 7463) | (5275; 8133) | (2822; 9678) |
| 2015/2016 | (72; 1382) | (371; 938) | (104; 2339) | (1753; 3737) | (512; 2125) | (2042; 4585) | (2659; 6351) | (5718; 14672) |
| 2016/2017 | (-1380; -335) | (-423; 35) | (-2420; -670) | (-142; 1543) | (-237; 1082) | (5234; 7284) | (4638; 7740) | (2902; 10105) |
| 2017/2018 | (-1147; 297) | (-178; 378) | (-1334; 979) | (1160; 3256) | (41; 1903) | (1446; 4237) | (1844; 6061) | (2693; 11776) |

### Table S7. Ninety-five percent confidence intervals of estimated number of influenza-associated excess pneumonia or influenza, respiratory, and respiratory or cardiovascular deaths by age group and epidemic season in Portuguese public hospitals between 2008/2009 and 2017/2018

| Season | Age groups (in years)  95% confidence interval | | | | | | | |
| --- | --- | --- | --- | --- | --- | --- | --- | --- |
|  | 0-4 | 5-18 | 19-49 | 50-64 | 65-74 | ≥75 | ≥65 | All ages |
| P&I influenza-associated deaths | | | | | | | | |
| 2008/2009 | -^a^ | (-1; 2) | (4; 14) | (3; 18) | (-1; 25) | (325; 484) | (332; 505) | (335; 532) |
| 2009/2010 |  | (-1; 36) | (20; 29) | (-23; -6) | (-36; -10) | (-317; -147) | (-350; -163) | (-357; -148) |
| 2010/2011 |  | (0; 2) | (3; 13) | (-3; 15) | (-12; 15) | (-111; 74) | (-116; 87) | (-110; 107) |
| 2011/2012 |  | (0; 2) | (6; 15) | (-9; 9) | (64; 89) | (788; 966) | (858; 1053) | (850; 1058) |
| 2012/2013 |  | (0; 2) | (8; 19) | (-4; 15) | (3; 35) | (119; 340) | (122; 363) | (144; 396) |
| 2013/2014 |  | (-1; 2) | (12; 20) | (-7; 9) | (-21; 5) | (-44; 149) | (-57; 146) | (-44; 172) |
| 2014/2015 |  | (-1; 4) | (9; 17) | (37; 51) | (44; 67) | (727; 885) | (770; 943) | (831; 1006) |
| 2015/2016 |  | (3; 5) | (23; 33) | (39; 59) | (9; 41) | (3; 228) | (6; 249) | (91; 330) |
| 2016/2017 |  | (0; 2) | (-3; 6) | (22; 39) | (52; 79) | (570; 755) | (622; 817) | (642; 839) |
| 2017/2018 |  | (-1; 1) | (17; 27) | (40; 59) | (37; 70) | (586; 821) | (634; 892) | (687; 940) |
| Respiratory influenza-associated deaths | | | | | | | | |
| 2008/2009 | (3; 6) | (0; 3) | (21; 36) | (5; 33) | (57; 102) | (715; 973) | (747; 1040) | (739; 1071) |
| 2009/2010 | (-1; 2) | (2; 4) | (60; 73) | (20; 44) | (-2; 43) | (-437; -139) | (-443; -114) | (-369; 2) |
| 2010/2011 | (4; 7) | (3; 6) | (35; 51) | (67; 95) | (37; 86) | (-78; 237) | (-37; 308) | (76; 449) |
| 2011/2012 | (-3; 1) | (2; 5) | (11; 25) | (-6; 22) | (119; 165) | (1315; 1619) | (1465; 1805) | (1482; 1850) |
| 2012/2013 | (-3; 2) | (-1; 3) | (19; 36) | (24; 56) | (-29; 32) | (83; 449) | (74; 478) | (156; 580) |
| 2013/2014 | (-1; 2) | (-1; 2) | (22; 34) | (17; 47) | (-36; 13) | (-156; 168) | (-188; 165) | (-100; 271) |
| 2014/2015 | (-1; 2) | (0; 2) | (13; 24) | (70; 95) | (118; 163) | (1395; 1675) | (1500; 1805) | (1608; 1924) |
| 2015/2016 | (-1; 3) | (2; 5) | (34; 50) | (48; 82) | (15; 73) | (-161; 234) | (-173; 256) | (-58; 386) |
| 2016/2017 | (-2; 1) | (-1; 2) | (9; 21) | (32; 59) | (84; 134) | (1053; 1383) | (1097; 1469) | (1127; 1496) |
| 2017/2018 | (1; 5) | (-2; 2) | (17; 33) | (38; 79) | (104; 162) | (930; 1339) | (1027; 1493) | (1059; 1496) |
| R&C influenza-associated deaths | | | | | | | | |
| 2008/2009 | (3; 6) | (0; 4) | (13; 47) | (47; 115) | (183; 274) | (1548; 2181) | (47; 115) | (1904; 2680) |
| 2009/2010 | (-2; 2) | (1; 5) | (70; 100) | (16; 80) | (-43; 48) | (-725; -136) | (16; 80) | (-750; 4) |
| 2010/2011 | (3; 7) | (4; 8) | (28; 63) | (82; 155) | (33; 132) | (-262; 467) | (82; 155) | (44; 900) |
| 2011/2012 | (-3; 3) | (0; 4) | (2; 38) | (-63; 12) | (79; 175) | (1838; 2549) | (-63; 12) | (1851; 2677) |
| 2012/2013 | (-3; 2) | (-2; 4) | (-26; 16) | (-35; 51) | (-137; -16) | (73; 958) | (-35; 51) | (-6; 1003) |
| 2013/2014 | (0; 4) | (-1; 3) | (57; 88) | (-21; 51) | (-108; -7) | (-832; -87) | (-21; 51) | (-855; 2) |
| 2014/2015 | (0; 3) | (1; 4) | (20; 48) | (122; 184) | (236; 320) | (2695; 3325) | (122; 184) | (3185; 3869) |
| 2015/2016 | (-1; 3) | (1; 6) | (34; 72) | (54; 144) | (35; 154) | (-1166; -272) | (54; 144) | (-1043; -62) |
| 2016/2017 | (-1; 2) | (1; 5) | (-1; 31) | (76; 149) | (143; 244) | (2248; 2950) | (76; 149) | (2541; 3319) |
| 2017/2018 | (0; 5) | (-4; 1) | (-31; 11) | (-27; 74) | (235; 363) | (984; 1953) | (-27; 74) | (1198; 2243) |

a. Not estimated due to the low number of deaths in this age group
